# Thermally Controlled Charge‐Carrier Transitions in Disordered PbSbTe Chalcogenides

**DOI:** 10.1002/adma.202106868

**Published:** 2021-11-19

**Authors:** Valentin Evang, Johannes Reindl, Lisa Schäfer, Alexander Rochotzki, Pauline Pletzer‐Zelgert, Matthias Wuttig, Riccardo Mazzarello

**Affiliations:** ^1^ Institute for Theoretical Solid State Physics RWTH Aachen University 52056 Aachen Germany; ^2^ I. Institute of Physics (IA) RWTH Aachen University 52056 Aachen Germany; ^3^ JARA‐FIT and JARA‐HPC RWTH Aachen University 52056 Aachen Germany; ^4^ Peter Grünberg Institute (PGI 10) Forschungszentrum Jülich GmbH 52428 Jülich Germany; ^5^ Department of Physics Sapienza Università di Roma Piazzale Aldo Moro 2 Roma 00185 Italy

**Keywords:** charge‐carrier transition, defect‐formation energies, density‐functional theory, lead antimony telluride, metavalent bonding

## Abstract

Binary and ternary chalcogenides have recently attracted much attention due to their wide range of applications including phase‐change memory materials, topological insulators, photonic switches, and thermoelectrics. These applications require a precise control of the number and mobility of charge carriers. Here, an unexpected charge‐carrier transition in ternary compounds from the PbTe–Sb_2_Te_3_ pseudo‐binary line is reported. Upon thermal annealing, sputtered thin films of PbSb_2_Te_4_ and Pb_2_Sb_2_Te_5_ undergo a transition in the temperature coefficient of resistance and in the type of the majority charge carriers from n‐type to p‐type. These transitions are observed upon increasing structural order within one crystallographic phase. To account for this striking observation, it is proposed that the Fermi energy shifts from the tail of the conduction band to the valence band because different levels of overall structural disorder lead to different predominant types of native point defects. This view is confirmed by an extensive computational study, revealing a transition from excess cations and Sb_Pb_ for high levels of disorder to Pb_Sb_ prevailing for low disorder. The findings will help fine‐tune transport properties in certain chalcogenides via proper thermal treatment, with potential benefits for memories, thermoelectric material optimization, and neuromorphic devices.

## Graphical abstract

It is shown experimentally that the carrier type and mobility in Pb–Sb–Te thin films can be controlled by annealing the disordered crystal. First‐principles simulations indicate that the unexpected transition from n‐ to p‐conduction is caused by a crossover of the predominant point‐defect type with increasing order. Tuning self‐doping properties this way could prove useful for thermoelectric devices and other applications.

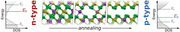

## 1 Introduction

Many crystalline chalcogenides have attracted attention in the past decade due to their unusual physical properties and bonding mechanism.^[^ ^1^, ^2^, ^3^, ^4^, ^5^, ^6^ ^]^ For many applications ranging from phase‐change memory devices^[^ ^7^, ^8^, ^9^ ^]^ and photonic switches^[^ ^10^, ^11^, ^12^ ^]^ over thermoelectric devices^[^ ^13^, ^14^, ^15^, ^16^, ^17^ ^]^ to prototype devices exploiting topological effects,^[^ ^18^, ^19^, ^20^ ^]^ the ability to tailor the electrical transport, for example, by varying the stoichiometry or by annealing, is of crucial importance. In particular, control of the charge‐carrier concentration and mobility would be highly advantageous. For instance, for devices based on the conducting surface states of topological insulators, it is generally important to eliminate unwanted sources of bulk carriers in order to suppress bulk transport. As regards thermoelectric devices, both n‐type and p‐type materials with carefully controlled carrier concentration are required. Efforts in these directions have concerned, for example, the chemical tuning of carrier types in a series of ternary tellurides^[^ ^21^, ^22^ ^]^ and the observation of Anderson transitions induced by thermal annealing in GeSbTe (GST) compounds (such as, Ge_2_Sb_2_Te_5_) and similar disordered chalcogenides.^[^ ^23^, ^24^, ^25^, ^26^, ^27^ ^]^ These chalcogenides lie on the tie‐line between a IV–VI and a V_2_VI_3_ material (e.g., GeTe and Sb_2_Te_3_ for GST). In the former case,^[^ ^22^ ^]^ stoichiometry variations were used to induce a transition from electron‐ to hole‐dominated charge transport, while in the latter case,^[^ ^23^, ^24^, ^25^, ^26^, ^27^ ^]^ the stoichiometry was kept constant and the level of disorder was tuned by annealing a crystalline phase, leading to an insulator‐metal transition upon increasing order.

Amorphous GST crystallizes into a metastable, disordered, rock‐salt‐like phase with Te occupying the anion sites and Ge, Sb, and vacancies randomly occupying the cation sites. The stable, hexagonal phase is obtained by further annealing the cubic structure. The three phases are semiconducting, but the crystalline states display a high concentration of p‐type carriers due to self‐doping effects, that is, due to native point defects that cause conductive, bulk‐like states to be occupied by holes and shift the Fermi energy toward the valence‐band maximum. This phenomenon leads to a strong electrical contrast between the amorphous and crystalline phase, which is exploited in phase‐change memories, where fast transitions between the two states are induced by proper electrical pulses.^[^ ^2^ ^]^

Analogous compounds containing Pb instead of Ge (PST) have mostly been observed and examined in their stable, hexagonal phase so far.^[^ ^28^, ^29^, ^30^ ^]^ An exception are nano‐crystals that have been found in a rock‐salt‐like structure.^[^ ^31^ ^]^

Here, we show that it is possible to grow PST in the rock‐salt‐like phase, and we thoroughly characterize the resulting metastable structure. We find that the carrier concentration and hence the transport characteristics can be tuned in a controlled fashion by annealing, that is, increasing the order of the atomic arrangement. Remarkably, PST even exhibits a transition from n‐type to p‐type doping upon increasing order, that is, without a change in composition. Density‐functional theory (DFT) simulations explain the transition on the basis of native point defects and a crossover of their formation energies, which vary as a function of overall structural disorder. The ability to change the dominant defect upon annealing and achieve both n‐ and p‐type conduction in PST could be useful for thermoelectric devices, which require both n‐ and p‐type legs. It is often preferred that both types of legs are made from the same material because of compatibility issues.

## 2 Results

### 2.1 Experiments

**Figure 1:**
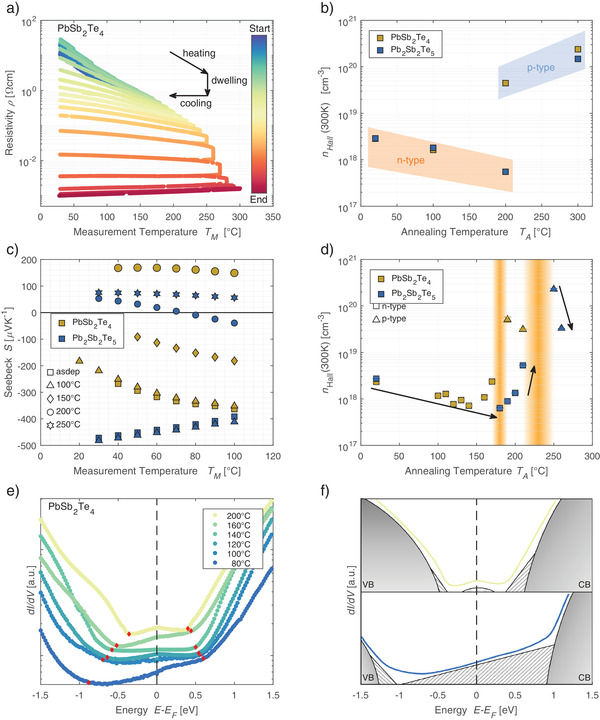
a) The resistivity of PbSb_2_Te_4_ is measured in a van‐der‐Pauw geometry. The sample is heated to a target temperature where it dwells and is subsequently cooled down to room temperature. For low annealing temperatures, the initial resistivity at 30 °C increases. At higher annealing temperatures, the resistivity decreases with increasing annealing temperature. b) The carrier concentration measured with an AC‐Hall setup decreases for increasing annealing temperatures in the n‐type regime. At 200 °C, PbSb_2_Te_4_ exhibits p‐type conduction while Pb_2_Sb_2_Te_5_ still is n‐type. c) The Seebeck coefficient for the as‐deposited phase and the samples annealed at lower temperatures is negative. For increasing annealing temperatures, the Seebeck coefficient changes sign and, for Pb_2_Sb_2_Te_5_ annealed to 200 °C, crosses the zero line. d) The carrier concentration at 300 K measured in a Hall‐bar geometry shows that the transition temperature for the carrier‐type transition is lower for PbSb_2_Te_4_ than for Pb_2_Sb_2_Te_5_. e) The tunneling density of states at 2 K for PbSb_2_Te_4_ shows that the Fermi level is situated in the tail of the conduction band. A visual guide of the evolution of the bands is given by the red dots, depicting a shift of the valence band to the Fermi level, while the band tail of the conduction band is reduced. f) The two sketches show how the DOS for the most and least annealed samples is decomposed into valence/conduction band and the disorder‐induced tail states (hatched area).

Inspired by the stoichiometric formulas of the well‐studied phase‐change materials GeSb_2_Te_4_ and Ge_2_Sb_2_Te_5_, thin films of PbSb_2_Te_4_ and Pb_2_Sb_2_Te_5_ are deposited from stoichiometric targets. Scanning electron microscopy images show a very smooth surface without any cubic structure, indicating that the polycrystalline films are not composed of nano‐crystals like those obtained previously.^[^ ^31^ ^]^ We carry out heated resistance measurements in a van‐der‐Pauw configuration, in which the material is cyclically annealed toward the phase‐segregation temperature. **Figure** **1** shows a series of transport measurements for PbSb_2_Te_4_. In Figure 1a, an exemplary resistivity measurement for PbSb_2_Te_4_ is depicted. It is apparent that, starting from the as‐deposited state, the resistance increases for lower annealing temperatures up to a maximum, whereas at higher annealing temperatures the resistance decreases and the temperature coefficient of the resistance (TCR) eventually changes from non‐metallic (TCR < 0) to metallic (TCR > 0) behavior. For chalcogenides with a large number of intrinsic vacancies, the decrease in resistance and the TCR sign change are common and are associated with the vacancy ordering process in the lattice and a concomitant reduction of disorder.^[^ ^23^, ^24^ ^]^ However, the initial increase in resistance cannot be explained by this mechanism, as the degree of disorder should decrease with annealing from the very beginning.

In order to further investigate this phenomenon, the carrier concentration of samples in the van‐der‐Pauw configuration is measured with a custom‐designed AC‐Hall measurement setup, where the samples have been annealed in a tube furnace and are subsequently characterized by X‐ray diffraction (XRD). Figure 1b shows that the charge‐carrier type of the specimen annealed at low temperatures is n‐type. The concentration of these charge carriers decreases for increasing annealing temperatures. Upon a further raise of annealing temperature, there is a transition to p‐type conduction, with a carrier concentration that increases with increasing annealing temperature. The carrier‐type transition occurs for PbSb_2_Te_4_ at lower annealing temperatures than for Pb_2_Sb_2_Te_5_. At the maximum temperature of 300 °C, the charge‐carrier densities are not only governed by the PST phases but also influenced by the emerging PbTe and Sb_2_Te_3_ phases (see structural analysis below), leading to a carrier density close to the value for Sb_2_Te_3_.

The change of the predominant carrier type is also confirmed by Seebeck measurements as shown in Figure 1c. Both PbSb_2_Te_4_ and Pb_2_Sb_2_Te_5_ reveal a negative value of the Seebeck coefficient *S* in the as‐deposited phase and for low annealing temperatures. The sign changes with increasing annealing temperature, confirming the presence of a carrier‐type transition. In the n‐type regime, the increase of |*S*| with temperature for PbSb_2_Te_4_ indicates that the Fermi‐level is situated in the conduction band, while Pb_2_Sb_2_Te_5_ shows a decreasing |*S*| upon increasing temperature compatible with the Fermi level lying outside this band. An interesting feature of the measurement is the presence of a sign change of the Seebeck coefficient for Pb_2_Sb_2_Te_5_ as a function of measurement temperature (for a fixed annealing temperature of 200 °C), which cannot be explained within a single‐band picture. At least a second band with different carrier type and mobility has to be present to account for the sign change in the coefficient. Further measurements of the carrier concentration in a Hall‐bar geometry with a better resolution of the annealing temperature confirm the carrier‐type transition as shown in Figure 1d. The strong growth in carrier concentration at the transition point again requires the presence of two‐band transport. As for the Seebeck measurements, the transition temperature is lower for PbSb_2_Te_4_ than for Pb_2_Sb_2_Te_5_.

An additional means to characterize the transition is to scan the density of states (DOS) for increasing annealing temperatures. To this end, the derivative of the tunneling current with respect to voltage, which is proportional to the DOS, is measured at 2 K. The resulting spectra for PbSb_2_Te_4_ are shown in Figure 1e. At the lowest annealing temperature, a pronounced band tail reaching from the conduction‐band edge into the band gap can be identified leading to a finite DOS at the Fermi level. The transition from majority n‐type to p‐type conduction can be connected to the decrease of this tail state with annealing while the valence‐band edge simultaneously moves closer to the Fermi energy, leading to the reduction of the band gap. This transition is sketched in the diagrams in Figure 1f, where the decomposition of the DOS into the valence/conduction bands and the disorder states is depicted. A similar scenario is present in the DOS data of Pb_2_Sb_2_Te_5_, which can be found in Figure S1, Supporting Information. A general shift in the band tails can be observed for both alloys, where the valence band tends to become more prominent for higher annealing temperatures.

**Figure 2:**
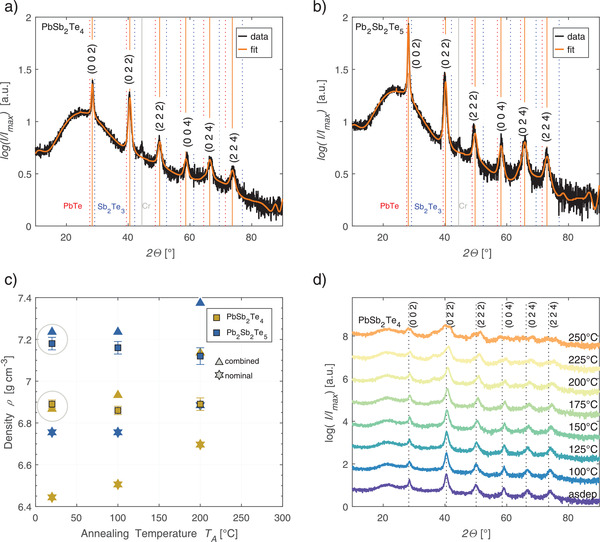
XRD measurements of the as‐deposited films of a) PbSb_2_Te_4_ and b) Pb_2_Sb_2_Te_5_. The peaks, marked by orange lines, exhibit the cubic symmetry of the crystal. Their positions do not coincide with peaks that would be expected for PbTe (dashed red lines) or Sb_2_Te_3_ (dashed blue lines) in their cubic configuration. The amorphous background of the scan is caused by the glass substrate. c) The density measured with XRR (squares) is compared to densities derived from the lattice parameter and the nominal (star) composition as well as from the composition obtained experimentally by combining information from EDX and RBS (triangle). d) For increasing annealing temperatures, no transition to the hexagonal phase can be observed in PbSb_2_Te_4_. However, the peak shifts indicate a small decrease of the lattice constant.

These peculiar transport properties hint toward more intricate structural changes in Pb*_x_* Sb*_y_* Te*_z_*‐alloys than in GST, where p‐type self‐doping is always observed. Thus, we thoroughly characterize the structure and composition of the Pb*_x_* Sb*_y_* Te*_z_* films by XRD experiments; see **Figure** **2**. The films possess a rock‐salt‐like structure. Comparing the measured XRD patterns to the theoretical positions for a perfect cubic structure (see Table S1, Supporting Information), small shifts of certain peaks from these positions are indicative of minor distortions from the ideal, rock‐salt‐like phase. The small size of these distortions is also evidenced by the absence of considerable peak splittings, which, instead, are common for other metastable phases of PCMs.^[^ ^32^, ^33^ ^]^

To check the elemental compositions obtained, electron‐dispersive X‐ray spectroscopy (EDX) measurements are performed on the films. The experiments are supplemented (for PbSb_2_Te_4_ only) by Rutherford backscattering spectrometry (RBS) to refine the elemental fractions, which are challenging to measure accurately in thin films with EDX since the penetration depth of the electrons is much larger than the film thickness. **Table** **1** gives the atomic compositions and the fraction of intrinsic vacancies on the cation sublattice, estimated from the results of each of the two experimental methods. EDX measurements reveal an excess of Pb and Sb as compared with their ideal fractions dictated by the nominal stoichiometry of each compound. We attribute this excess to additional Pb and Sb atoms residing on cation sites with the Te sublattice being fully occupied, an assumption which will be substantiated by additional data below. The results of RBS measurements are then used to pin the fraction of Pb in the overall crystal, yielding 16% in PbSb_2_Te_4_, to be compared with the fraction of Pb in stoichiometric PbSb_2_Te_4_ (14.3% Pb). Combining these results, we assume considerably more Pb and Sb atoms than expected from the nominal composition. These excess atoms can occupy vacant lattice sites, leading to a decreased number of intrinsic, cationic vacancies. This assumption is supported by measurements of the film density with X‐ray reflectometry (XRR) as shown in Figure 2c: The average density of films deposited with different working pressures exceeds the nominal density, which is derived from the XRD lattice constant assuming an ideal, rock‐salt‐like lattice containing 25% and 20% vacancies on the cation sites in PbSb_2_Te_4_ and Pb_2_Sb_2_Te_5_, respectively. Assuming an increased Pb and Sb content and a decreased vacancy content, the EDX‐ and RBS‐corrected values agree with the XRR densities to about 0.3% and 0.8% for PbSb_2_Te_4_ and Pb_2_Sb_2_Te_5_, respectively. Also for intermediate annealing temperatures (100 °C), at which the XRR‐measured density of Pb_2_Sb_2_Te_5_ appears to slightly decrease, its value remains in better agreement with the prediction based on the XRD lattice constant if excess cations are included in the stoichiometry.

**Table 1:** Stoichiometries and vacancy contents on the cation sublattice of the as‐deposited films of PbSb_2_Te_4_ and Pb_2_Sb_2_Te_5_

	PbSb_2_Te_4_				Pb_2_Sb_2_Te_5_			
	Stoichiometry			Vacancies	Stoichiometry			Vacancies
Nominal	1	2	4	25.0%	2	2	5	20.0%
EDX‐corrected	1.11	2.22	4	16.6%	2.13	2.13	5	14.8%
RBS‐corrected	1.14	2	4	21.5%	2.28	2	5	14.4%
Combined	1.14	2.28	4	14.5%	2.28	2.28	5	8.8%

The EDX‐corrected values are based on the atomic ratios measured by EDX and on the assumption that the anion sublattice is fully occupied by Te atoms. Additionally, RBS measurements were performed on PbSb_2_Te_4_ to determine the exact Pb fraction in the sample, since this method is more precise than EDX for thin films. The corresponding values for Pb_2_Sb_2_Te_5_ are obtained from those for PbSb_2_Te_4_ by assuming an equal relative deviation of the Pb content per compositional unit. The bottom row combines the results from EDX (twice as many Sb as Pb atoms) with the precise number of Pb atoms from RBS (1.14 Pb atoms per 4 cationic lattice sites).

Further annealing experiments of newly generated samples on SiO_2_ capped with (ZnS)_80_:(SiO_2_)_20_, conducted in an Ar protective gas atmosphere, exhibit additional XRD peaks for higher annealing temperatures (250–300 °C); see Figure S2, Supporting Information. These peaks are not associated with the cubic phase of the alloys but stem from a separation into hexagonal Sb_2_Te_3_ and rock‐salt‐like PbTe. No transition to hexagonal PST is observed. Although grazing incidence XRD measurements on thin films generally do not allow a quantitative evaluation of the peak intensities, the more pronounced intensity of the peaks associated with PbTe in Pb_2_Sb_2_Te_5_ compared to PbSb_2_Te_4_ strengthens the hypothesis of phase separation into the constituents, since a higher PbTe content in Pb_2_Sb_2_Te_5_ is expected.

### 2.2 Simulations

The suggested path of the Fermi energy from conduction‐band to valence‐band‐related states calls for a theoretical explanation. The conduction properties of a semiconductor are generally influenced not only by dopants (i.e., impurities) but also by native point defects, which can cause conductive, bulk‐like states to be occupied by electrons (or holes), accompanied by a shift of the Fermi energy toward the conduction‐band minimum (or the valence‐band maximum). This phenomenon is called self‐doping. In fact, numerous point defects are expected in the as‐deposited and low‐annealed specimens. It is thus insightful to examine point defects in disordered PST and estimate which defect types should prevail at the various stages of annealing.

To achieve this goal, we compute formation energies of intrinsic defects in the disordered and in the ordered, cubic phase. The latter represents the maximally ordered structure with cubic stacking, consisting of pure layers of either Pb, Sb, or vacancies perpendicular to the [111] direction, alternating with pure Te layers.

12 possible point defects are considered: Vacancies (V_Pb_, V_Sb_, V_Te_); additional atoms occupying empty sites of the cation sublattice (Pb_V_, Sb_V_, Te_V_); exchanges within the group of cationic atoms (Pb_Sb_, Sb_Pb_); and anti‐site defects (Pb_Te_, Sb_Te_, Te_Pb_, Te_Sb_). Interstitials (Pb_i_, Sb_i_, Te_i_), that is, additional atoms located in the center of a cube of four cation and four anion sites, are found to be considerably more expensive than the corresponding defects (X_V_) on vacant lattice sites (approximate difference for Pb: +2 eV, Sb: +1.5 eV, Te: +1 eV). This confirms the assumption previously made that excess Pb and Sb atoms in the deposited films occupy the cationic sublattice. Since the multitude of geometrically different structures in the disordered systems requires a large number of calculations, we confine the scope of this study to charge‐neutral systems, in line with two works reporting on defect‐formation energies (DFEs) for disordered, rock‐salt‐like GST.^[^ ^34^, ^35^ ^]^ The conceptual link to the type of charge carrier resulting from a defect is instead established on the basis of the chemical role that group IV/V elements and group VI elements typically assume in chalcogenide compounds. For example, Pb_Sb_ leads to one electron missing in the local bond network and thus to p‐type conduction. On the other hand, V_Te_ can be interpreted to describe the lack of one “backbone” Te atom, permitting the electrons provided by surrounding cationic atoms to contribute to n‐type conduction. In other words, the highest valence‐band states in these chalcogenides consist mostly of Te p‐orbitals, so that more valence‐band states than electrons are removed together with a Te atom. Indeed, V_Te_ was shown to act as a donor defect in GeTe^[^ ^36^ ^]^ and PbTe.^[^ ^37^ ^]^ In total, the 6 defects that lead to n‐type conduction are V_Te_, Pb_V_, Sb_V_, Sb_Pb_, Te_Pb_, and Te_Sb_.

We perform DFT simulations^[^ ^38^, ^39^ ^]^ and adopt the supercell method to compute the formation energy of a defect.^[^ ^40^, ^41^, ^42^ ^]^ For the disordered systems, a large number of independent models is generated to guarantee statistically significant energies. For ordered systems, defects on Te sites are computed for all inequivalent lattice sites, i.e., on sites next to vacancy layers (Te1), between Pb and Sb layers (Te2), and between two Pb layers (Te3, in Pb_2_Sb_2_Te_5_ only). More information can be found in the Computational Details section.

DFEs depend on the chemical potential of each element involved in producing the defect, that is, on the energetic level of the reservoir from which an atom is removed or in which it is deposited. These elemental reference energies are expressed as

(1)

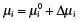

where *µ_i_* ^0^ are the DFT total energies. The negative Δ*µ_i_* are interrelated to obey the stability of the host material and are further restricted by secondary phases that could be formed along with the host.^[^ ^42^ ^]^

**Figure 3:**
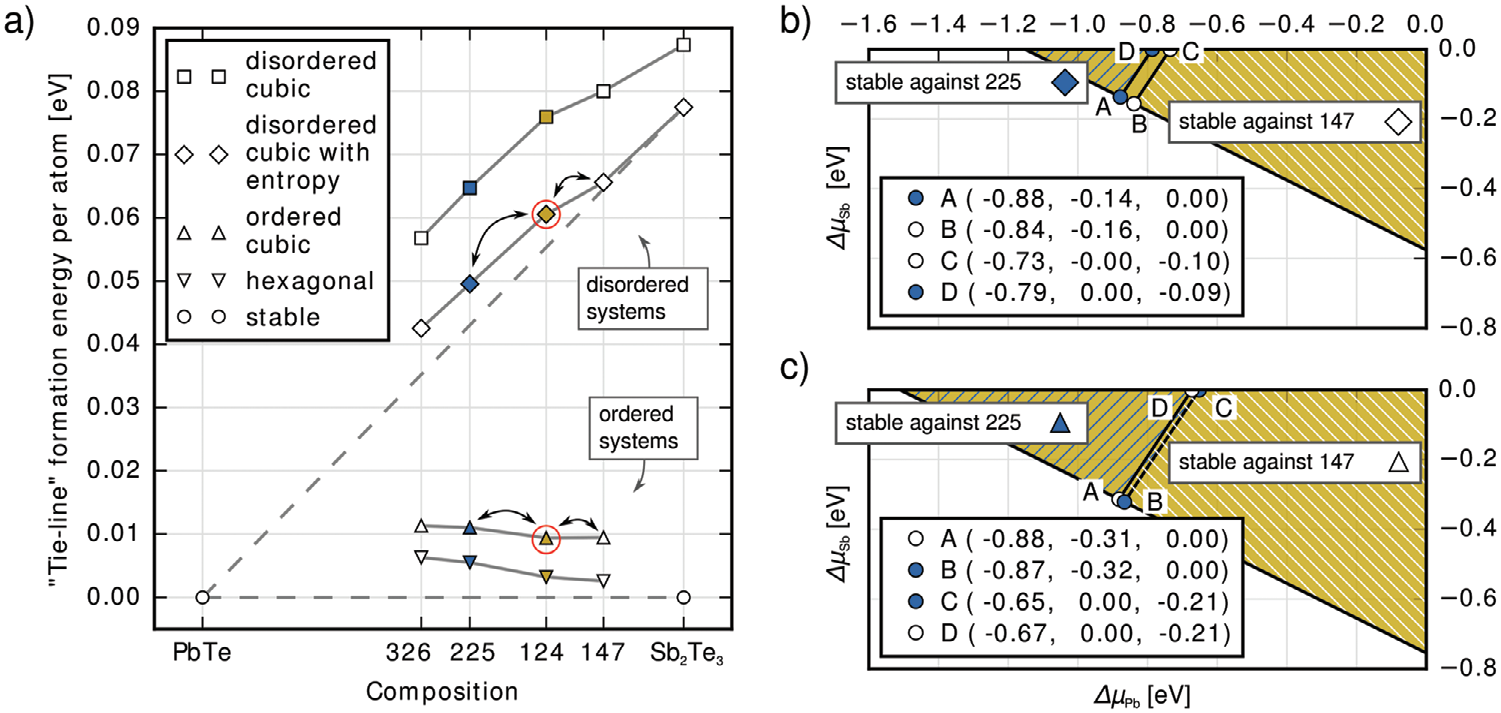
a) Phase‐formation energies of various PST compounds in their ordered and disordered structures with respect to their stable “parent materials” PbTe and Sb_2_Te_3_. The stability limit of compounds between PbTe and the ordered, hexagonal and disordered, cubic phases of Sb_2_Te_3_ is represented by dashed lines: Interestingly, even the hexagonal structures are slightly unstable with respect to the stable, binary compounds. Disordered, cubic systems have very high formation energies, but they remain only slightly unstable if disordered, rock‐salt‐like Sb_2_Te_3_ is taken as a reference (diagonal, dashed line). Materials analyzed in parts b and c of the figure are marked with red circles. To define growth conditions Δ*µ_i_*, only close‐by compounds are considered, as indicated by black double arrows. b) Regions of stability for disordered PbSb_2_Te_4_ with respect to Pb_2_Sb_2_Te_5_ and PbSb_4_Te_7_. The Δ*µ*_Te_‐axis points toward the reader. The coordinates in the box are the chemical potentials of Pb, Sb, and Te (expressed as Δ*µ_i_*) at the four limiting points. The regions of stability do not overlap, in accordance with the slight instability visible in part a. c) Regions of stability for ordered PbSb_2_Te_4_ with respect to Pb_2_Sb_2_Te_5_ and PbSb_4_Te_7_. Here, a small region exists where PbSb_2_Te_4_ is stable against decomposition.

Formation energies of various PST phases with respect to PbTe and Sb_2_Te_3_ are shown in **Figure** **3**a. For the disordered structures, the random occupation of sites on the cation sublattice is accounted for by including configurational entropy at 300 K, corresponding approximately to the growth temperature of the films. As explained in a previous study on defects in GST,^[^ ^35^ ^]^ assuming simultaneous equilibria of a disordered compound with the stable binary phases (namely, PbTe and Sb_2_Te_3_ for PST) to define extremal growth conditions is not possible because of the high energy of formation; in fact, doing so would not even reflect the out‐of‐equilibrium situation of the metastable phase. Instead, we assume equilibria with those phases that are very similar to the host in terms of stoichiometry and structure and can therefore be expected to represent the actual reservoirs needed for atomic exchange. We follow the same approach for the ordered host materials.

Figure 3b shows the regions of stability of disordered PbSb_2_Te_4_. Because of the slight instability against decomposition into PbSb_4_Te_7_ and Pb_2_Sb_2_Te_5_, there is no common region of overall stability. On the other hand, considering the very process of defect formation in the host, choosing chemical potentials along, for example, the line A–D would correspond to an exchange of particles with a just coexisting Pb_2_Sb_2_Te_5_ phase. By interpreting points on this line as reference energies, we assume a partial equilibrium with one of the neighbor phases (Pb_2_Sb_2_Te_5_) but stay as close as possible to the region of stability with the other neighbor phase (PbSb_4_Te_7_). Figure 3c shows the thin, proper region of stability and derived growth conditions for ordered, cubic PbSb_2_Te_4_. The results for Pb_2_Sb_2_Te_5_ can be found in Figure S3, Supporting Information.

**Figure 4:**
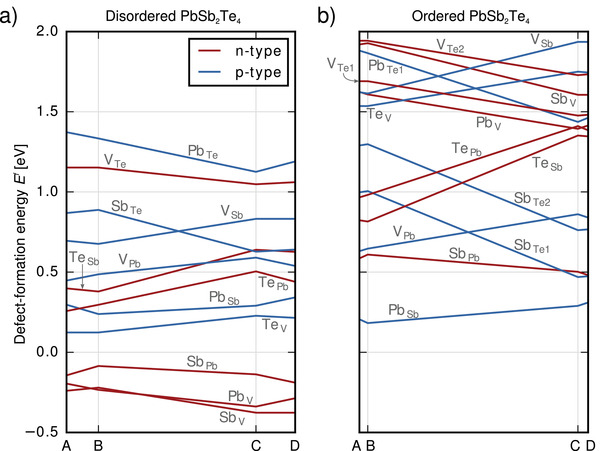
Defect‐formation energies in disordered and ordered PbSb_2_Te_4_ as a function of the growth conditions corresponding to the paths A–B–C–D in chemical‐potential space defined in Figure 3b,c. For instance, A and B denote Te‐rich conditions (Δ*µ*_Te_ = 0), making defects that comprise additional Te atoms relatively cheap. In the disordered, cubic phase, Sb_Pb_, Pb_V_, and Sb_V_ are the most favorable defects, leading to n‐doping. On the contrary, Pb_Sb_ has the lowest formation energy in the ordered structure, yielding p‐doping. This finding is independent from the growth conditions. In the ordered structure, defects on Te sites occur two times each due to inequivalent lattice sites (Te1: next to vacancy layer; Te2: between Sb and Pb layers).

DFEs for PbSb_2_Te_4_ and Pb_2_Sb_2_Te_5_ are shown in **Figure** **4** and Figure S4, Supporting Information, respectively. Different growth conditions are considered for each phase by walking along the paths A–B–C–D in chemical‐potential space as defined in Figure 3b,c (Figure S3a,b, Supporting Information, for Pb_2_Sb_2_Te_5_).

The principal observations made for the relevant, low‐energy defects are the same for both stoichiometries. In disordered PST, the three defects Sb_V_, Pb_V_, and Sb_Pb_ have the lowest formation energies. With energies around −0.3 to −0.5 eV, it is especially favorable to insert additional Sb and Pb atoms on intrinsic vacancies. Thus, three defects associated with n‐type conductivity are the easiest to form in the disordered phase. In the ordered phase, on the other hand, formation energies are considerably larger for most defects. This is especially true for additional atoms on intrinsic vacancies, including Sb_V_ and Pb_V_: While these defects are among the most favorable ones in disordered PST, their formation energies are around and above 1.5 eV in the ordered, cubic phase. Also the third type with negative formation energy in disordered PST, Sb_Pb_, becomes much less favorable when turning to the ordered structure as it undergoes an increase by 0.7–0.9 eV.

Regarding p‐type defects, the additional Te atom occupying an empty site (Te_V_) and the Pb atom replacing an Sb atom (Pb_Sb_) are the least expensive in the disordered phase, but have formation energies that are positive for all growth conditions and are much less favorable than the three n‐type defects discussed above: Even when considering the chemical potentials at point B, which most strongly penalize the addition of Sb while favoring the addition of Te, the energy difference between Te_V_ and Sb_V_ is still greater than 0.3 eV for PbSb_2_Te_4_ and greater than 0.4 eV for Pb_2_Sb_2_Te_5_.

On the other hand, in the ordered phase, the p‐type defect Pb_Sb_ becomes the lowest‐energy defect, in spite of the fact that its formation energy is comparable to the one in disordered PST. As a result, a crossover of the most favorable defects from such related to n‐type conductivity to one associated with p‐type conductivity (Pb_Sb_) has occurred upon increasing the order in the lattice. The ordering process also brings about the change in sign of the TCR.^[^ ^23^, ^24^, ^27^ ^]^ The energy difference to the lowest defect associated with n‐type conductivity, Sb_Pb_, is about 0.2–0.4 eV. Hence, in ordered PST, defects associated with p‐type conductivity are predominant in the low‐energy region below 1 eV over the whole range of reference chemical potentials.

## 3 Discussion

The experiments show that nominal bulk PbSb_2_Te_4_ and Pb_2_Sb_2_Te_5_ exhibit an excess of Pb and Sb in their as‐deposited, disordered state. This is in line with DFT calculations revealing negative formation energies for Pb_V_ and Sb_V_. The Te vacancy and interstitials are found to be much more expensive, justifying the assumption that only defects on the cation sublattice lead to this stoichiometric deviation.

The predominance of excess cations (probably combined with the presence of Sb_Pb_ defects) is furthermore responsible for the n‐type conduction observed for low annealing temperatures in Hall and Seebeck measurements. We note that the specific values of the DFEs should be treated with caution since the interpretation of growth conditions is not straightforward due to the metastability of the disordered phase. In addition, simulations that go beyond neutral defects could be helpful to confirm the type and number of charge carriers produced. Nevertheless, we believe that the principal energetic trends among the different defect types are well captured in the simulations, especially because of the very low formation energies of Pb_V_, Sb_V_, and Sb_Pb_. Interestingly, DFT simulations of disordered GST^[^ ^35^ ^]^ yield negative DFEs for the analogous defect types, too, in contradiction with the experimentally observed p‐type conduction in these compounds. However, the energetic advantage of these n‐type defects is small enough in GST to be lifted by a band‐gap correction argument. Here, in PST, the large energetic difference to the lowest‐lying p‐type defects is expected to be robust against errors associated with the DFT band‐gap problem. This point is further discussed in the Supporting Information (“Remark on band‐gap corrections in GST and PST”).

Given the unexpected, initial increase in resistance, we conclude that the transport properties as a function of annealing are, in the n‐type regime, more strongly governed by the diminishing carrier concentration than by the carrier mobility, which generally increases upon structural ordering. The latter process, in combination with multi‐band effects, explains the transition to the p‐type regime characterized by higher carrier concentrations and lower resistivity. For Pb_2_Sb_2_Te_5_, the influence of two bands with different carrier types even leaves a fingerprint in the Seebeck coefficient changing its sign as a function of the measurement temperature. For PbSb_2_Te_4_, on the other hand, the changing relevance of conduction‐band and valence‐band tails over the course of annealing can best be tracked in tunneling measurements of the DOS. Altogether, the experiments imply that the Fermi level must shift downward with respect to the band extrema upon structural ordering. The differences between PbSb_2_Te_4_ and Pb_2_Sb_2_Te_5_ in the Seebeck data suggest that the two processes involved—reduction of band tails and Fermi‐energy shift—concur in different ways in the two materials, with Pb_2_Sb_2_Te_5_ starting already with a comparatively low Fermi energy but yet noticeable band tails.

On the atomistic level, the DFT study reveals Pb_Sb_ to be responsible for the p‐type conduction at higher structural order. One of the reasons that Pb_Sb_ becomes the dominant defect is that excess atoms on intrinsic vacancies (Pb_V_ and Sb_V_) become very expensive in the ordered structure since intrinsic vacancies form perfect layers and reduce the Te–Te interlayer distance.

The second ingredient for Pb_Sb_ becoming the relevant defect is the strong increase in formation energy of the competing defect Sb_Pb_ upon increasing order. Sb sites in the ordered structures are close to vacancy layers, which provide much room and relatively weak bonds for effective relaxation. Pb sites, on the other hand, are surrounded by at least three atomic layers in each direction. The significance of this effect is confirmed by a fictional, hexagonal model of Pb_2_Sb_2_Te_5_ with swapped Sb and Pb layers: Compared with the true structure, Sb_Pb_ profits more from the swap than Pb_Sb_ does by 0.3 eV, equaling approximately the difference in formation energy of these defects in the ordered phase. Due to entropic effects, a certain degree of compositional Pb/Sb disorder could be present even in the samples with ordered vacancy layers: this ingredient is not included in our models. Nevertheless, experiments indicate that the cation layers close to vacancy layers are predominantly occupied by Sb atoms,^[^ ^29^ ^]^ which corroborates our explanation above.

Following our argument about the expected charge states, the simulations hence explain an n‐type to p‐type charge‐carrier transition on the basis of specific, intrinsic point defects: While in disordered PST, the dominant defects are such that lead to a surplus of valence electrons with respect to the average of three p‐electrons per lattice site, the most favorable defect in the ordered phase, Pb_Sb_, entails a lack of valence electrons.

**Figure 5:**
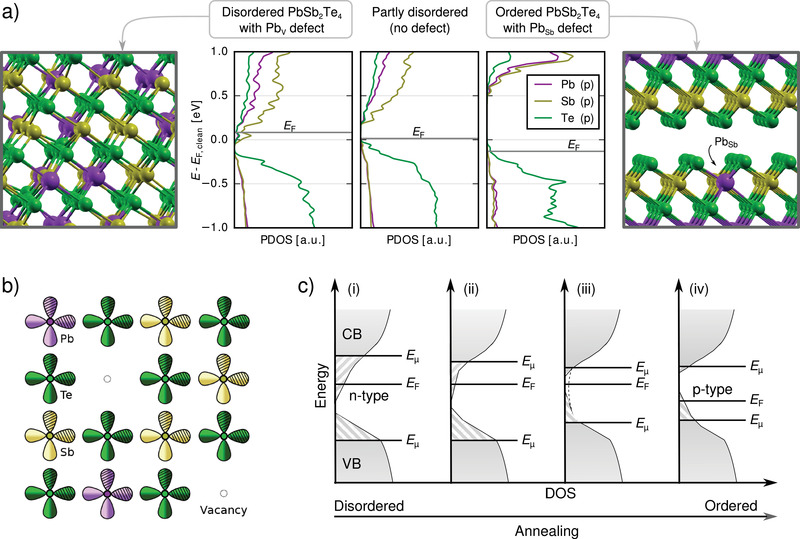
a) Density of states of various models projected onto atomic p‐orbitals (PDOS). An additional Pb atom in the disordered phase leads to n‐doping. At intermediate levels of disorder on the cation sublattice, the band edges become slightly sharper than in the fully disordered case; with no defects present, the Fermi energy E_F_ lies inside the band gap. The Pb_Sb_ defect in the ordered phase shifts E_F_ down into the valence band (p‐doping). The PDOS curves are shifted vertically to align the Te s‐bands with those of the defect‐free, disordered system, whose E_F_ is used as the zero of energy. b) A 2D arrangement of atoms, representing the xy‐plane of rock‐salt‐like PST, with two p‐valence‐orbitals per atom indicated and two vacant cation sites. c) Toy model explaining the observed carrier transition upon thermal annealing of disordered, rock‐salt‐like PST. The as‐deposited phase i) corresponds to the fully disordered, rock‐salt‐like structure shown in (b). As the material becomes more ordered upon annealing, the band tails become less pronounced i–iii). Concurrently, a transition from n‐ to p‐doping takes place ii–iv), which is due to the crossover of predominant point defects as a function of structural order. Pb, Sb, and Te atoms are drawn in purple, yellow, and green, respectively. Part b: Reproduced with permission.^[^ ^27^ ^]^ Copyright 2021, Wiley‐VCH

The shift of the Fermi energy and the carrier transition as a result of ordering and a crossover of defects is reproduced in **Figure** **5**a showing the DOS projected onto atomic p‐orbitals. In the disordered system, the exemplary Pb_V_ defect indeed shifts the Fermi energy into the conduction band, which has smeared‐out tails due to the high degree of disorder. In the ordered system, on the other hand, the Pb_Sb_ defect leads to a lowered Fermi energy and hole conduction. The data also supports the fact that mainly Te p‐orbitals constitute states at the valence‐band maximum, whereas states at the conduction‐band minimum are principally composed of cation p‐orbitals.

In the following, we present a model of the electronic structure capturing the various effects observed in the experiments and simulations; see Figure 5b,c. The aim of the model is to explain both the decrease and subsequent increase of the number of charge carriers and the transition from n‐type to p‐type conduction. The valence p‐orbitals of Pb, Sb, and Te atoms (depicted in Figure 5b) form a network of half‐filled σ‐bonds in the rock‐salt‐like phase. Peierls‐like distortions from the perfect structure open a small band gap. Nevertheless, rock‐salt‐like PST shows n‐type conductivity (Figure 5c–i) due to excess Pb and Sb atoms sitting on intrinsic vacancies in the mixed cation planes, as well as to Sb_Pb_ defects. Furthermore, PST is characterized by high values of the Born effective charge and the static dielectric constant: These are fingerprints of metavalent bonding, a peculiar bonding mechanism stemming from the competition between electron localization and delocalization.^[^ ^15^, ^16^, ^43^ ^]^ The high dielectric constant leads to a suppression of correlation effects, which, in combination with the strong vacancy disorder, yields disorder‐induced localization.^[^ ^27^ ^]^

The reduction of charge carriers upon annealing in a regime of activated transport is due to the conduction‐band tail providing fewer and fewer states around the Fermi energy as a direct result of a thermally induced ordering process (i)–(ii). That the resistivity starts to decrease before the carrier density reaches its minimum can be interpreted to be caused by a decrease of the conduction‐band mobility edge: While the number of thermally activated charge carriers goes down, their mobility increases. Finally, the carrier‐type transition must be due to the Fermi energy passing through a critical point in the band gap from above (iii) and approaching the valence‐band maximum (iv). As the material is still observed to be in an activated‐transport regime, the Fermi energy must still lie above the valence‐band mobility edge, but it is expected to be close to conductive valence‐band states given the strongly increased carrier concentrations. Only when annealed beyond the carrier‐transition temperature does the material become metallic.

In conclusion, we have grown PST compounds in the disordered, metastable phase and have observed a charge‐carrier transition from n‐type to p‐type upon annealing before any transition to a different crystal structure takes place. From DFT calculations, we have found an explanation for the charge‐carrier transition on the basis of a crossover of prevailing point defects from Pb_V_, Sb_V_, and Sb_Pb_ (before annealing) to Pb_Sb_ (after annealing).

From the theoretical point of view, the strongly disordered n‐doped phase is particularly interesting, since the microscopic mechanisms responsible for electron localization in p‐doped rock‐salt‐like chalcogenides,^[^ ^24^, ^27^ ^]^ namely the vacancy clusters, cannot account for Anderson‐insulating behavior in n‐doped materials. Further work is needed to pin down the relevant mechanism.

Regarding applications, our work offers a new path for tailoring electronic properties based on Fermi‐level tuning via annealing. Many thermoelectric materials are low‐bandgap semiconductors; thus, bipolar conduction due to excitation of minority carriers often decreases their thermoelectric performance. The change in type of predominant defects and the consequent change in majority carrier concentration reported in this work could be useful to suppress such bipolar effects. At the same time, care must be taken that the maximum operating temperature of the device does not exceed the transition temperature of the material.

We expect that the mechanism proposed for the transition from p‐doping to n‐doping can occur in other binary or ternary chalcogenides as long as the following properties are obeyed: a) At low annealing temperature, the alloy can form a disordered, rock‐salt‐like phase with large concentration of stoichiometric vacancies in one sublattice; b) in this phase, the energetically favorable defects are excess cations sitting in this sublattice; c) annealing at higher temperature induces a transition to an ordered, layered phase, which cannot accommodate the excess atoms. Recently, we carried out a systematic computational screening over binary and ternary chalcogenides in the rock‐salt‐like phase, which yielded a stability map based on simple chemical indicators.^[^ ^27^ ^]^ This map enables compounds obeying property (a) to be identified. Furthermore, property (c) is quite generic, since the disordered, rock‐salt‐like phase is typically metastable. By exploring the map, one can pin down materials that exhibit property (b) and determine optimal candidates in terms of carrier mobility. Thus, independent control over doping type and mobility should be achievable, opening up new possibilities for tailoring electronic states for thermoelectrics, topological materials, and phase‐change materials for memories and neuromorphic devices.

## 4 Experimental Section

Thin‐film specimens were grown by DC‐magnetron sputtering from stoichiometric targets. The samples used for XRD measurements in Figure 2a,b, density measurements in Figure 2c, resistance measurements in Figure 1a, and carrier concentration measurements in Figure 1b–d have been deposited with pd‐factors (product of working pressure and target distance) from 1.95e‐2 mBarcm to 3.10e‐2 mBarcm for Pb_2_Sb_2_Te_5_ and 1.95e‐2 mBarcm to 3.21e‐2 mBarcm for PbSb_2_Te_4_. The samples characterized with XRD and electrical means were deposited in a van‐der‐Pauw configuration with shadow masks, with a 1 cm^2^ active square area on a glass substrate with Cr bottom contacts at the edges and a 15 nm (ZnS)_80_:(SiO_2_)_20_ capping layer. For the DOS measurements, tunnel junctions in a crossbar structure were sputtered in‐situ with Al contacts and a 1.5 nm reactively sputtered Al_2_O_3_ layer as a tunnel barrier. The XRR samples have been deposited on Si(100) without a capping layer. The samples for the temperature‐dependent XRD measurements in Figure 2d and Figure S2a, Supporting Information, the Seebeck samples, and the carrier‐concentration measurements in Figure 1d were deposited with a pd‐factor of 3.15e‐2 mBarcm for PbSb_2_Te_4_ and 2.25e‐2 mBarcm for Pb_2_Sb_2_Te_5_. The substrate was Si(100) with 1 µm SiO_2_ capping for the XRD and carrier‐concentration measurements and Corning 1737 glass for the Seebeck measurements. The film thickness for all films was ≈80 nm.

The XRD and XRR measurements have been performed in a Philips X'pert Analytical X‐ray setup. The measurement setups for the resistance, the AC‐Hall, and Seebeck measurements are custom built. The carrier concentration in the Hall bar geometry was measured with a PPMS and a Dynacool from Quantum Design. The DOS at 2 K was determined by tunneling spectroscopy with an Ametek Lock‐in Amplifier and a Keithley SMU, which were connected to the PPMS.

### Computational Details

The formation energy *E^f^* [*X*] of a defect *X* was obtained within the supercell approach and DFT according to^[^ ^40^, ^41^, ^42^ ^]^

(2) $$E^{f}\left[X\right]=E_{\text{tot}}\left[X\right]-E_{\text{tot}}\left[\text{bulk}\right]-\underset{i}{\sum }n_{i}\mu_{i}$$

Here, *E*_tot_[*X*] and *E*_tot_[bulk] denote the total ground‐state energy of the supercell with and without the point defect, respectively. The reference chemical potential of element *i* is given by *µ_i_*, and *n_i_* is the number of atoms of element *i* added to the pristine cell to generate the defect (so *n_i_* < 0 if atom *i* is removed). Pristine, that is, defect‐free cells were relaxed to their theoretical densities, whereas only atomic positions were relaxed after introducing the defects while keeping the simulation box fixed.

Models of disordered PbSb_2_Te_4_ and Pb_2_Sb_2_Te_5_ contain 189 and 194 atoms, respectively, the latter being a computationally feasible compromise yielding approximately Pb_1.99_Sb_1.99_Te_5_. To guarantee statistically significant energies, 16 independent pristine models were generated for each stoichiometry, with cationic atoms randomly placed on their sublattice. For each defect type and each of the 16 “parent” models, eight lattice sites were chosen to generate an instance of the defect, leading to 128 computed instances per defect type and stoichiometry. The supercells of ordered PbSb_2_Te_4_ and Pb_2_Sb_2_Te_5_ contain 336 and 432 atoms, respectively.

Total‐energy calculations and relaxations were done using the plane‐wave code implemented in the Quantum Espresso suite of programs.^[^ ^44^ ^]^ We used the Perdew–Burke–Ernzerhof exchange‐correlation functional^[^ ^45^ ^]^ in combination with the semi‐empirical DFT‐D3 dispersion correction^[^ ^46^ ^]^ to account for the relevance of long‐range interaction in elemental Te and ordered alloys. Ultrasoft (Sb, Te) and norm‐conserving pseudopotentials (Pb) were employed with a wave‐function cutoff of 30 Ry leading to energy convergence below 2 meV per atom in the elemental phases. For disordered systems, a 2 × 2 × 2 k‐point mesh shifted away from the Γ point was used to obtain energy convergence below 1 mRy per atom and to avoid the sampling of extrema of spurious defect‐level bands. For the ordered systems, a shifted 2 × 2 × 1 k‐point mesh was used.

## Conflict of Interest

The authors declare no conflict of interest.

## Supporting information

Supporting Information

## Data Availability

Research data are not shared.
