# Supplementary material for: Thermally Controlled Charge‐Carrier Transitions in Disordered PbSbTe Chalcogenides
Source: Adv Mater. 2021 Nov 19;34(3):2106868. doi: 10.1002/adma.202106868 (PMC11469063; doi:10.1002/adma.202106868)
Supplement: Supplementary file 1 — Supporting Information [file ADMA-34-2106868-s001.pdf]

ADVANCED MATERIALS

Supporting Information

for *Adv. Mater.,* DOI: 10.1002/adma.202106868

Thermally Controlled Charge-Carrier Transitions in Disordered PbSbTe Chalcogenides

Valentin Evang, Johannes Reindl, Lisa Schäfer, Alexander Rochotzki, Pauline Pletzer-Zelgert, Matthias Wuttig, and Riccardo Mazzarello**

Supporting Information

Thermally Controlled Charge-Carrier Transitions in Disordered PbSbTe Chalcogenides

V. Evang, J. Reindl, L. Schäfer, A. Rochotzki, P. Pletzer-Zelgert, M. Wuttig, R. Mazzarello

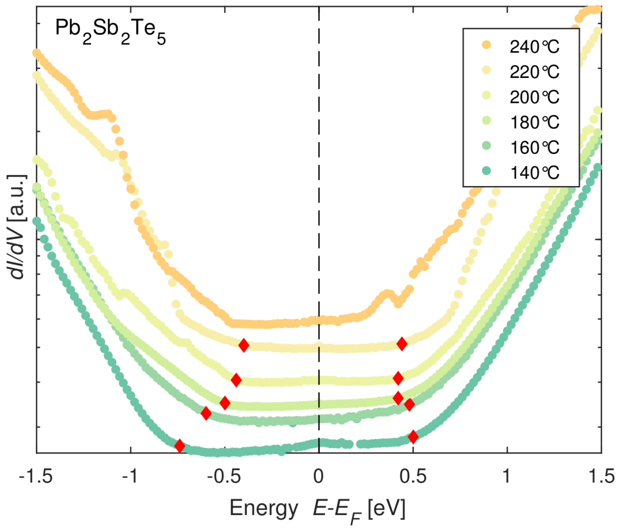

Figure S1: DOS for $Pb_2Sb_2Te_5$. The shift of the valence band (left side) towards the Fermi energy as well as the reduction of the conduction-band tail are observed. The samples annealed at 240 °C show signs of trap states, with distinct peaks in the conduction and valence band.

Table S1: Measured XRD peak positions compared to the positions for an ideal cubic system. Small deviations indicate a slight deviation from the perfect rock-salt-like structure.

	$PbSb_2Te_4$		$Pb_2Sb_2Te_5$	
	2θ (°)		2θ (°)	
hkl	Data	Theory	Data	Theory
(0 0 2)	28.48	28.38	28.17	28.12
(0 2 2)	40.43	40.54	39.97	40.20
(2 2 2)	50.05	50.22	49.43	49.76
(0 0 4)	59.03	58.62	58.26	58.12
(0 2 4)	66.58	66.42	65.80	65.80
(2 2 4)	73.92	73.66	72.93	73.00

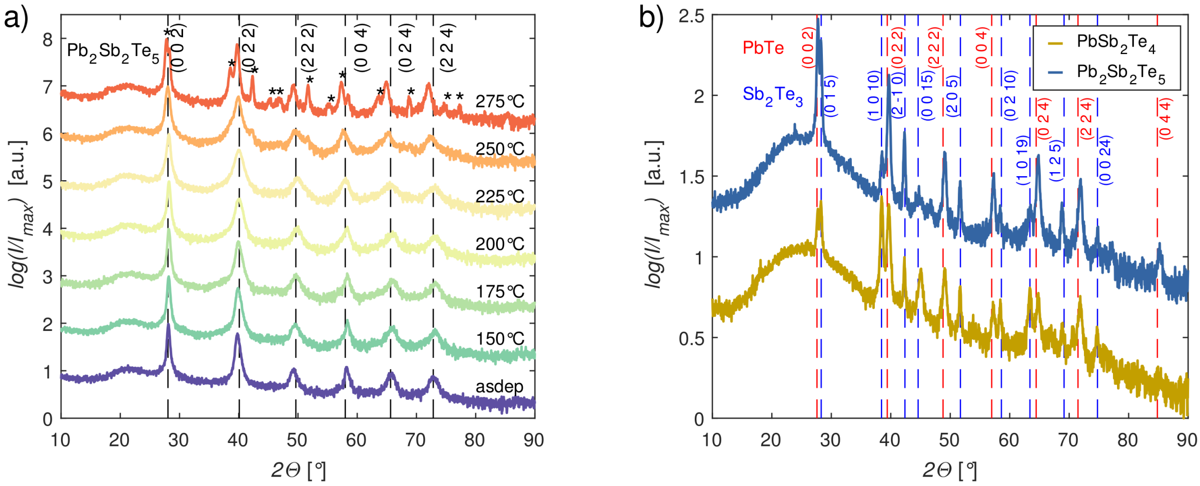

Figure S2: XRD spectra after annealing. a) Additional peaks not associated with the rock-salt-like phase can be identified for highest annealing temperatures in $Pb_2Sb_2Te_5$. b) At 300 °C, for both $PbSb_2Te_4$ and $Pb_2Sb_2Te_5$, the lattice separates into the constituents with peaks associated with the cubic phase of PbTe in red and peaks associated with the hexagonal phase of Sb_2Te_3 in blue.

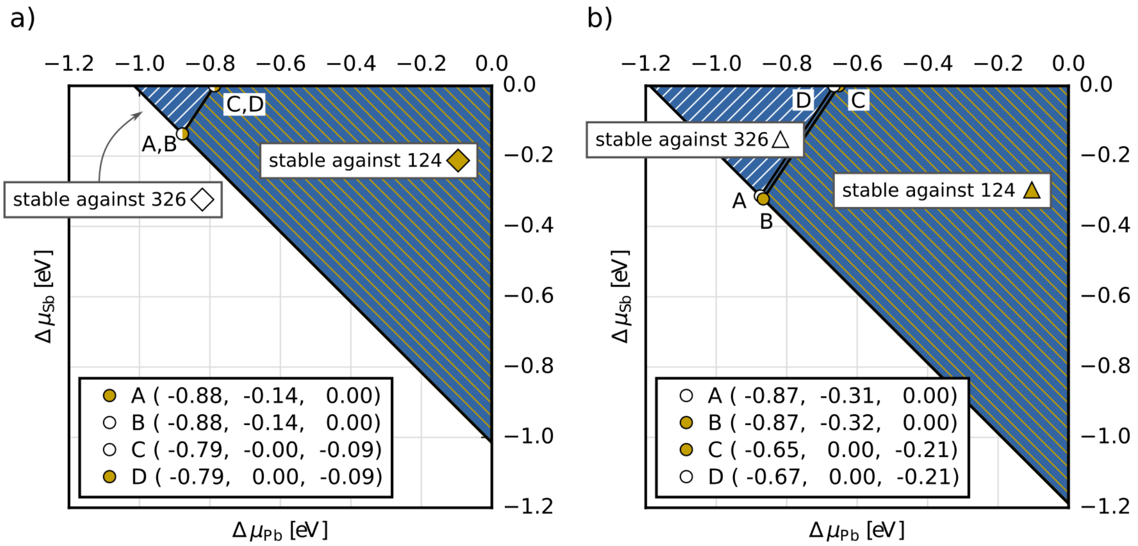

Figure S3: a) Regions of stability for *disordered* $Pb_2Sb_2Te_5$ (with configurational entropy) with respect to the stoichiometrically closest phases. The coordinates in the box are the chemical potentials of Pb, Sb, and Te (expressed as $\Delta\mu_i$) at the four limiting points. The regions of stability just touch each other, in accordance with the corresponding phase-formation energies lying on a straight line in Figure 3a. b) Regions of stability for *ordered* $Pb_2Sb_2Te_5$ with respect to the stoichiometrically closest phases. The areas just do not overlap.

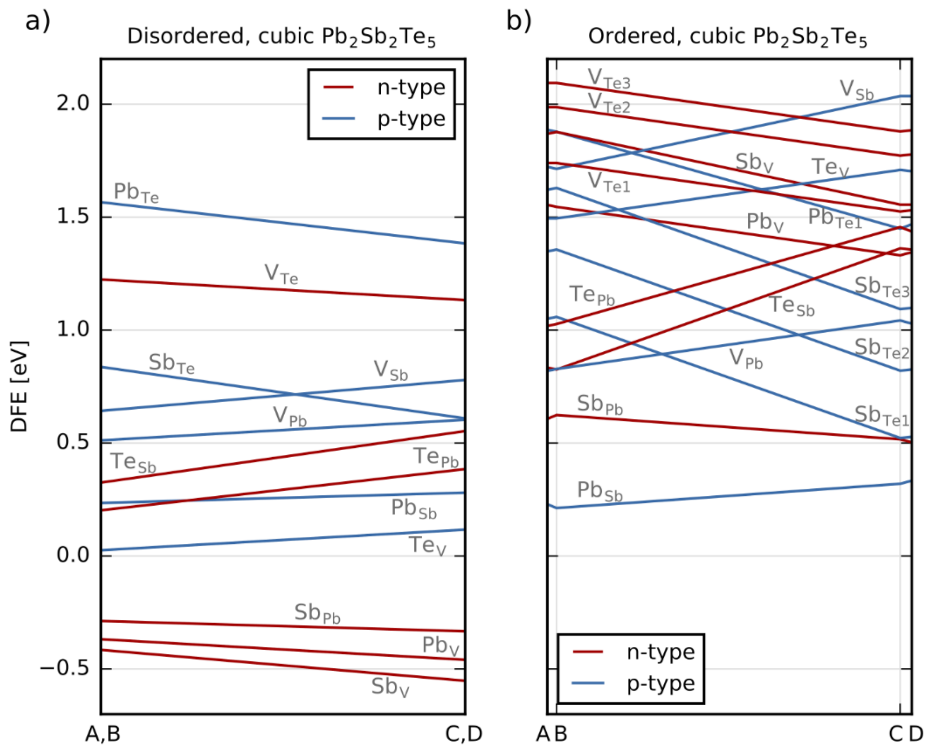

Figure S4: Defect-formation energies in disordered and ordered $Pb_2Sb_2Te_5$ as a function of the growth conditions corresponding to the paths A–B–C–D in chemical-potential space defined in Figure S3a and S3b. As for $PbSb_2Te_4$, in the disordered, cubic phase, Sb_{Pb}, Pb_V, and Sb_V are the most favorable defects, leading to n-doping. On the contrary, Pb_{Sb} has the lowest formation energy in the ordered structure, yielding p-doping. In the ordered structure, defects on Te sites occur three times each due to inequivalent lattice sites (Te1: next to vacancy layer; Te2: between Sb and Pb layers; Te3: between two Pb layers).

Remark on band-gap corrections in GST and PST

In disordered, cubic $GeSb_2Te_4$ and $Ge_2Sb_2Te_5$, our recent DFT study[1] reveals the point defects Sb_V, Ge_V, and Sb_{Ge} associated with n-doping to have the smallest formation energies. It was noted that misplaced band edges, which are common in DFT calculations, could erroneously lower the true formation energies, especially for defects associated with n-doping. Comparing the DFT gap to the experimental gap in GST, we found that a band-edge correction via shifting the conduction-band minimum lifts these defects by about 0.25 eV for each electron that is introduced into the bulk system. The formation energies of the n-type defects would then become comparable to those of certain p-type defects in the low-energy regime; thus, neither n- or p-doping is clearly suggested by the simulations alone. Combined with the experimentally observed p-type conduction, the Ge_{Sb} defect was identified to probably play the most important role for explaining p-doping in this material.[1]

In the present study of $PbSb_2Te_4$ and $Pb_2Sb_2Te_5$, an analogous argument proves more difficult because of the lack of studies reporting the corresponding experimental band gaps. Given the available values of 0.42 eV for nano-crystallites of disordered, cubic $Pb_2Sb_2Te_5$[2] and 0.48 eV for stable, hexagonal $PbSb_2Te_4$,[3] a rough estimate on the order of 0.5 eV seems appropriate. Comparing this with the HOMO-LUMO gap estimated as 0.25 eV for a disordered model of $PbSb_2Te_4$ leads to a band-gap correction of n-type defects of around 0.25 eV, i.e., similar to GST. However, in PST, the difference in energy between the lowest n-type defect and the lowest p-type defect is about 0.1–0.15 eV larger compared with GST, supporting the predominance of n-type defects even after band-edge corrections. We furthermore note that the upward shift of 0.25 eV is an upper limit for the correction of n-type defects; if a downward shift of the valence-band edge were considered to recover the true band gap, p-type defects would instead be lifted. Finally, the experiments presented here strongly support the finding that additional Pb and Sb atoms are the main defects in the disordered phase. This situation is drastically different from GST compounds, where an excess of cations is not experimentally observed.

References

[1] V. Evang, R. Mazzarello, *Materials Science in Semiconductor Processing* **2021**, *133*, 105948.

[2] R. B. Soriano, I. U. Arachchige, C. D. Malliakas, J. Wu, M. G. Kanatzidis, *Journal of the American Chemical Society* **2013**, *135*, 768.

[3] S. Talreja, B. Ahuja, *Journal of Materials Science* **2017**, *52*, 346.
